# Supplementary material for: Allelic diversity uncovers protein domains contributing to the emergence of antimicrobial resistance
Source: PLoS Genet. 2023 Mar 27;19(3):e1010490. doi: 10.1371/journal.pgen.1010490 (PMC10079234; doi:10.1371/journal.pgen.1010490)
Supplement: S1 Appendix — (PDF) [file pgen.1010490.s001.pdf]

```
>Wild-type_(N16961)
MDNKLGLNKMNKTLIALAVSAAAVATGAYADGINQSGDKAGSTVYSAKGTSLEVGGRAEA
RLSLKDGKAQDNSRVRLNFLGKAEINDSLYGVGFYEGEFTTNDQGKNASNNSLDNRYTYA
GIGGTYGEVTYGKNDGALGVITDFTDIMSYHGNTAAEKIAVADRVDNMLAYKGQFGDLGV
KASYRFADRNAVDAMGNVVTETNAAKYSDNGEDGYSLSAIYTFGDTGFNVGAGYADQDDQ
NEYMLAASYRMENLYFAGLFTDGELAKDVDYTGYELAAGYKLGQAAFTATYNNAETAKET
SADNFAIDATYYFKPNFRSYISYQFNLLDSDKVGKVASEDELAIGLRYDF
>Clade_1_(IRLE0074)
MDNKLGLNKMNKTLIALAVSAAAVATGAYADGINQSGDKAGSTVYSAKGTSLEVGGRAEA
RLSLKDGKAQDNSRVRLNFLGKAEINDSLYGVGFYEGEFTTADNGTKDNKGSLDNRYTYA
GIGGTYGEVTYGKNDGALGVITDFTDIMSYHGNTAAEKIAVADRVDNMLAYKGQFGDLGV
KASYRFADRNAVDAMGNVVAETNAAKYSDNGEDGYSLSAIYTFGDTGFNVGAGYADQDEQ
NEYMLAASYRMENLYFAGLFTDGELAKDVDYTGYELAAGYKLGQAAFTATYNNAETAKET
SADNFAIDATYYFKPNFRSYISYQFNLLDSDKVGKVASEDELAIGLRYDF
>Clade_2_(GBE0658)
MDNKLGLNKMNKTLIALAVSAAAVATGAYADGINQSGDKAGSTVYSAKGTSLEVGGRAEA
RLSLKDGKAQDNSRVRLNFLGKAEINDSLYGVGFYEGEFTTNDQGKNASNNSLDNRYTYA
GIGGTYGEVTYGKNDGALGVITDFTDIMSYHGNSAADKIAVADRVDNMLAYKGQFGDLGV
KASFRFADRTENTVTDKYEDNGKDGYSLSAIYAFGDTGFNVGAGYADQDEQNEYMLAASY
RMENLYFAGLFTDGEKAKDVDYTGYELAAGYKLGQAAFTATYNNAETAKETSADNFAIDA
TYYFKPNFRSYISYNFNLLDSDKVGKVASEDELAIGLRYDF
>Clade_3_(IRLE0181)
MDNKLGLNKMNKTLIALAVSAAAVATGAYADGINQSGDKAGSTVYSAKGTSLEIGGRAEA
RLSLKDGKAEDKSRVRLNFLGKAEINDSLYGIGFYEGEFTTADSTATKTDNGSDLDNRYT
YAGIGGTYGEVTYGKNDGALGVITDFTDIMSYHGNSAADKIAVADRVDNMLAYKGQFGDL
GVKASFRFADRTENTVTDKYEDNGKDGYSLSAIYAFGDTGFNVGAGYADQDEQNEYMLAA
SYRMENLYFAGLFTDGEKAKDVDYTGYELAAGYKLGQAAFTATYNNAETAKETSADNFAI
DATYYFKPNFRSYISYNFNLLDSDKVGKVASEDELAIGLRYDF
>Clade_4_(IRLE0079)
MDNKLGLNKMNKTLIALAVSAAAVATGAYADGINQSGDKAGSTVYSAKGTSLEIGGRAEA
RLSLKDGKSEDKSRVRLNFLGKAEINDSLYGIGFYEGEFTTADNTDGSELDNRYTYAGIG
GTYGEVTYGKNDGALGVITDFTDIMSYHGNSAADKIAVADRTDNMLAYKGQFGDLGVKAS
YRFADRDTSTGEFTDNKEDGYSLSAIYAFGDTGFNIGAGYADQNDNNEYMLAASYRMENV
YFGALFTDGEKNFNSKSNGNNSVVKGKFTGIQDYTGYELAAGYKLGQAAFTTTYNNAETA
NDTSANNVAIDATYYFKPNFRTYISYNFNLLDSGDKLGNSTVSKIDAEDELAIGLRYDF
>Clade_5_(IRLE0081)
MDNKLGLNKMNKTLIALAVSAAAVATGAYADGINQSGDKAGSTVYSAKGTSLEVGGRAEA
RLSLKDGKAQDNSRVRLNFLGKAEINDSLYGVGFYEGEFTTNDQGKNESNNSLDNRYTYA
GIGGTYGEVTYGKNDGALGVITDFTDIMSYHGNSAADKIAVADRVDNMLAYKGQFGDLGV
KASYRFADRVEGTGTNSGQYVDNGKDGYSLSAIYAFGDTGFNVGAGYADQDEQNEYMLAA
SYRMENLYFAGLFTDGEKAATNGDYTGYELAAGYKLGQAAFTTTYNNAETNDETSANNFA
IDATYYFKPNFRTYISYNFNMIDAGDVLGKVGGNGVATKIDAEDELAIGLRYDF
>Clade_6_(GBE0428)
MDNKLGLNKMNKTLIALAVSAAAVATGAYADGINQSGDKAGSTVYSAKGTSLEIGGRAEA
RLSLKDGKAEDKSRVRLNFLGKAEINDSLYGIGFYEGEFTTADSTATKTDNGSDLDNRYT
YAGIGGTYGEVTYGKNDGALGVITDFTDIMSYHGNSAADKIAVADRVDNMLAYKGQFGDL
GVKASFRFADRTENTVTDKYEDNGKDGYSLSAIYAFGDTGFNVGAGYADQDEQNEYMLAA
SYRMENLYFAGLFTDGEKAATNGDYTGYELAAGYKLGQAAFTTTYNNAETNDETSANNFA
IDATYYFKPNFRTYISYNFNMIDAGDVLGKVGGNGVATKIDAEDELAIGLRYDF
>Clade_7_(GBE0917)
MDNKLGLNKMNKTLIALAVSAAAVATGVNAGEIYNQDGASLTMGGRAEARLSLKDGKAED
KSRVRLNFLGKAEINDSLYGVGFYEGEFTTNDSVSYEDPEKNGSDLDNRYTYAGIGGTYG
EVTYGKNDGALGVITDFTDIMAYHGNSAADKIAVADRTDNMLAYKGQFGDLGVKASFRFA
DRKENKATDKFEDNGKDGYSLSAIYAFGDTGFSVGAGYADQDTQDQYMLAASYRMENLYF
```

AGLFTDGEKAATNGDYTGYELAAGYKLGQAAFTTTYNNAETNDETSTNKFAIDATYYFKP
NFRSYISYNFNMIDAGDVLGKVGGNGVATKIDAEDELAIGLRYDF
>Clade_8_(IRLE0082)
MDNKLGLNKMNKTLIALAVSAAAVATGVNAGEIYNQDGASLTMGGRAEARLSLQDGKADD
KSRVRLNFLGKVAINDSLYGVGFYEGQFETNDDGVNKTNNNLDNRYTYAGIGGTYGEVTY
GKNDGALGVITDFTDIMAYHGNSAAYKIAVADRVDNMLAYKGQFGDLGVKASYRFADRKD
GTGSNAGKFVDNDKDGYSLSAIYAFGDTGFNIGAGYADQDTQDQYMLAASYAIADFYFAG
SFVDGQEKPANAAKTDKTGYELAAKYTMGQAVFSTTYNYLESKSSGTKTDEADNFAIDAT
YYFKPNFRSYISYNFNLLDEDANKGITKAQAEDELAIGLRYDF
>Clade_9_(GBE1116)
MDNKLGLNKMNKTLIALAVSAAAVATGVNAGEIYNKDGASLTMGGRAEARLSLQDGKADD
KSRVRLNFLGKVAINDSLYGVGFYEGEFTTADNTDGSELDNRYTYAGIGGTYGEVTYGKN
DGALGVITDFTDIMAYHGATAVGESKLPVADRTDNMLAYKGQFGDLGVKASYRFADRKNV
AGKYEDNGLDGYSLSAVYAIGETGITLGAGYADQETQSQYMLAASYVISDFYFAGSFVDG
QDKPANQTKADLTGYELAAKYTMGQAVFSTTYNYLESKTSGTKADEADNFAIDATYYFKP
NFRSYISYNFNLLDEDANKGITKAQAEDELAIGLRYDF
>Clade_10_(GBE1194)
MDNKLGLNKMNKTLIALAVSAAAVATGVNAGEIYNQDGASLTMGGRAEARLSLKDGKADD
KSRVRLNFLGKVAINDSLYGVGFYEGEFTTADKGTTDEKGSLDNRYTYAGIGGTYGEVTY
GKNDGALGVITDFTDIMAYHGNSAAYKIAVADRVDNMLAYKGQFGDLGVKASYRFADRSA
DKDVNGDWNNAYTDNNADGYSLSAVYAIGETGITLGAGYADQDNQNTSADQYMLAASYTI
SDFYFAGTFVDGQVKEFNDKADLTGYELAAKYTMGQAVFSTTYNYLESKSSGTKTDEADN
FAIDATYYFKPNFRSYISYNFNLLDEDANKGITKAQAEDELAIGLRYDF
>Clade_11_(RIMD_2214396)
MDNKLGLNKMNKTLIALAVSAAAVATGVNAGEIYNQDGASLNMGGRAEARLSLKDGKADD
KSRVRLNFLGKVAINDSLYGVGFYEGEFTTNDQGEAKDGNLNNRYTYAGIGGTYGEVTYG
KNDGALGVITDFTDIMAYHGNSAAYKIAVADRVDNMLAYKGQFGDLGVKASYRFADRSAD
KNANGDWNNAYTDNNADGYSLSAVYAFGDTGFNIGAGYADQDTEDQYMLAASYAIADFYF
AGAFVDGQDKPANAAKTDKTGYELAAKYTMGQAVFSTTYNYLESKSSGTKTDEADNFAID
ATYYFKPNFRSYISYNFNLLDEDANKGITKAQAEDELAIGLRYDF
>Clade_12_(FORC_073)
MDNKLGLNKMNKTLIALAVSAAAVATGVNAGEIYNQDGASLNMGGRAEARLSLKDGKADD
KSRVRLNFLGKVAINDSLYGVGFYEGEFTTNDRDGQNNNLENRYTYAGIGGTYGEVTYGK
NDGALGVITDFTDIMAYHGATAVGESKLHVADRADNMLAYKGQFGDLGVKASYRFADRDV
NAGKFVDNNKDGYSLSAVYAFGDTGFNIGAGYADQDTQDQYMLAASYAIADFYFAGSFVD
GQDKPANAAKTDKTGYELAAKYTMGQAVFSTTYNYLESKSSGTKTDEADNFAIDATYYFK
PNFRSYISYNFNLLDEDANKGITKAQVEDELAIGLRYDF
>Clade_13_(IDH-06787)
MDNKLGLNKMNKTLIALAVSAAAVATGVNAGEIYNQDGASLTMGGRAEARLSLKDGKADD
KSRVRLNFLGKVAINDSLYGVGFYEGEFTTADSTATETDNGSDLDNRYTYAGIGGTYGEV
TYGKNDGALGVITDFTDIMAYHGENGTYKLAVADRVDNMLAYKGQFGDLGVKASYRFADR
DEDGKFVEGVSNDKFIDKDNDGYSLSAIYTFGDTGFNVGAGYADQDTADQYILAASYAFS
DFYFAGAFVDGQVKPANAAKRDKTGYELAAKYTMDQTVFTTTYNYQEEKTSGVKSDNADF
IAIDATYYFKPNFRSYVSYNFNLLDADKVGKTKAEDELAIGLRYDF
>Clade_14_(GBE1114)
MDNKLGLNKMNKTLIALAVSAAAVATGVNAGEIYNQDGASLTMGGRAEARLSLKDGKADD
KSRVRLNFLGKVAINDSLYGVGFYEGEFTTNDQGEAKDGNLNNRYTYAGIGGTYGEVTYG
KNDGALGVITDFTDIMAYHGANGTYKLAVADRVDNMLAYNGQFGDLGVKASYRFADRQES
VNSITKNEQDGYSLSAIYAFGDTGFNVGAGYADQDTADQYILAASYAFSDFYFAGAFVDG
QVKPANAAKTDKTGYELAAKYTMDQTVFTTTYNYQEEKTSGVKSDNADFIAIDATYYFKP
NFRSYVSYNFNLLDADKVGKTKAEDELAIGLRYDF
