# Supplementary material for: Allelic diversity uncovers protein domains contributing to the emergence of antimicrobial resistance
Source: PLoS Genet. 2023 Mar 27;19(3):e1010490. doi: 10.1371/journal.pgen.1010490 (PMC10079234; doi:10.1371/journal.pgen.1010490)
Supplement: S2 Appendix — (PDF) [file pgen.1010490.s002.pdf]

RESULTS

Bile-directed evolution of isogenic *ompU* mutant strains.

We previously proposed that OmpU from toxigenic strains evolved in the environment and serve as preadaptations to virulence in the context of the human host [1, 2]. Our results here further corroborate this scenario as there are numerous environmental non-toxigenic strains that encode alleles of OmpU conferring bile resistance (**Fig 1C**). To further determine whether the domains associated with the bile resistance phenotype evolve through direct exposure to bile (pathoadaptations) or as predicted, were acquired prior to exposure to host-associated antimicrobials, we performed *in vitro* evolution experiments by exposing the isogenic mutant strains encoding the allele $ompU^{GBE1114}$ and $ompU^{0000}$ to bile. Briefly, strains were successively passaged through 0.4% whole bile until they exhibited WT levels of resistance (**S7A Fig**). The entire exposed culture was maintained between passages to minimize drift. Based on our results, we observed a steady increase in survival as $ompU^{0000}$ was passaged through bile. On the other hand, $ompU^{GBE1114}$, steadily increased to an average survival of ~8% within the first four passages but then plateaued from passage 4 through 7, until a sharp rise after passage 8 to WT survival levels (**S7B Fig**). Analyses of the OmpU alleles of three of these evolved strains indicate that OmpU does not exhibit changes over time suggesting that compensatory mutations in the background of *V. cholerae* lead to this phenotype in the presence of 0.4% bile (**S7C Fig**). Future work should resolve whether this results might also be due to the stringency of the treatment (0.4% bile) or whether higher resolution (samples) are necessary in order

to indetify more nuanced changes in the population. Nonetheless, taken together our results suggest that toxigenic OmpU has evolved as a preadaptation to virulence.

MATERIALS AND METHODS

Bile-directed *in vitro* evolution. *In vitro* evolution experiments were performed using the modified protocol described by Levin-Reisman et al [3]. Briefly, overnight cultures were diluted (1:100) and cultured in LB until reaching mid-log phase (OD_{600nm}~0.5). Cultures were washed 2X with LB and were either resuspended in LB or LB + 0.4% whole bile. Cultures were incubated at 37˚C with aeration for 1hr. Aliquots of the treated and untreated samples were used for c.f.u. enumeration and the remainder of the treated samples were washed twice and resuspended in LB supplemented 0.004% whole bile (1/100 of whole bile concentration used). To minimize drift all the surviving cells were kept between passages. DNA was extracted from ancestral and evolved strains using QIAGEN-Gentra Puregene Yeast/Bact. Kit.

REFERENCES

1. Shapiro BJ, Levade I, Kovacikova G, Taylor RK, Almagro-Moreno S. Origins of pandemic Vibrio cholerae from environmental gene pools. Nat Microbiol. 2016; 2:16240.
2. Balasubramanian, D., et al., *Molecular mechanisms and drivers of pathogen emergence.* Trends Microbiol, 2022. **30**(9): p. 898-911.
3. Levin-Reisman, I., et al., *Antibiotic tolerance facilitates the evolution of resistance.* Science, 2017. **355**(6327): p. 826-830.
