# Supplementary material for: Allelic diversity uncovers protein domains contributing to the emergence of antimicrobial resistance
Source: PLoS Genet. 2023 Mar 27;19(3):e1010490. doi: 10.1371/journal.pgen.1010490 (PMC10079234; doi:10.1371/journal.pgen.1010490)
Supplement: S1 Fig — Gray boxes represent missing residues and black dots represent conserved residues. Highlighted in black boxes are identified regions of interest numbered 1–7. Green and purple dots represent alleles from Cluster 1 and Cluster 2 respectively. (PPTX) [file pgen.1010490.s003.pptx]

## Slide 1
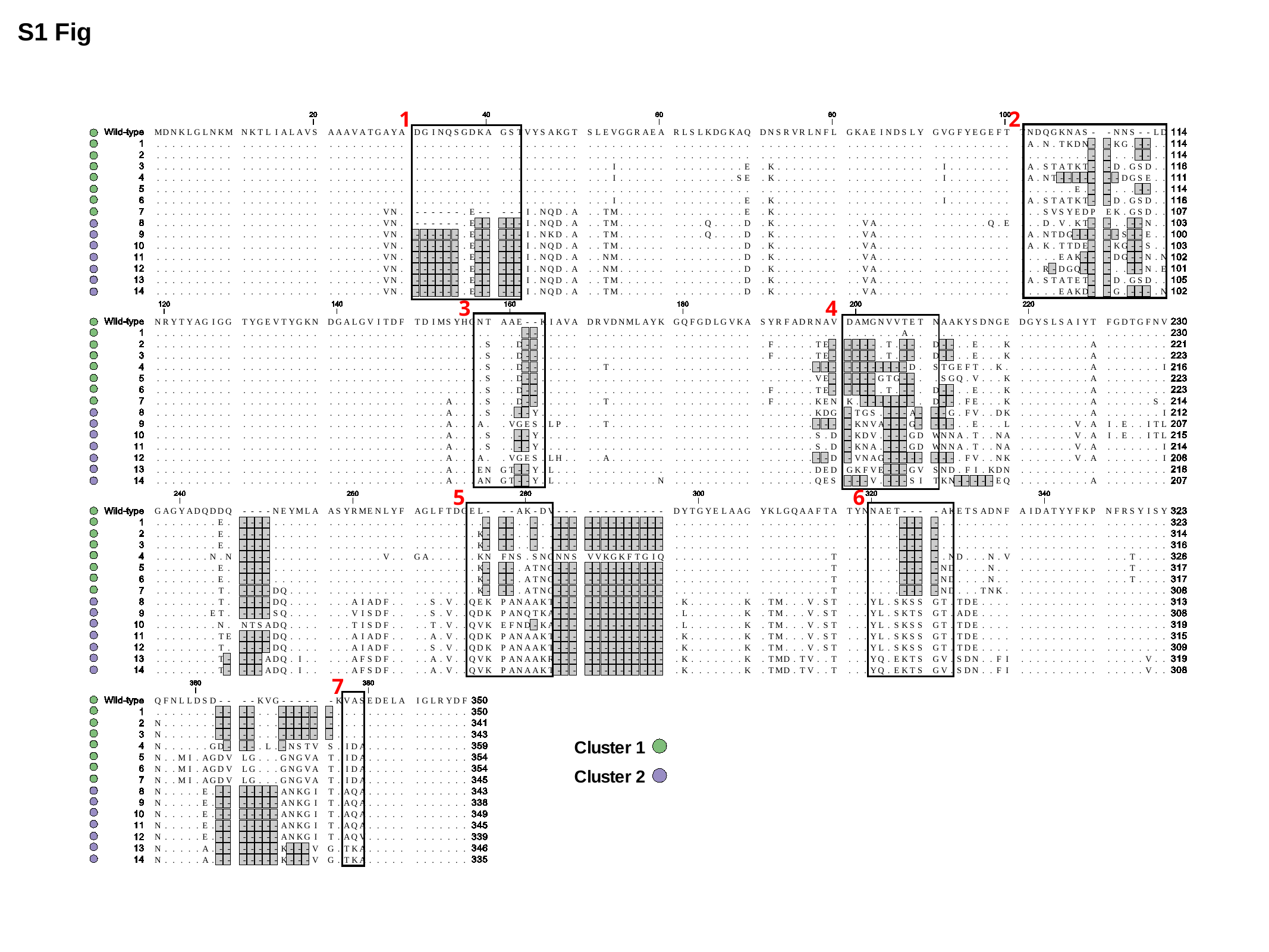

S1 Fig
1
2
M
D
N
K
L
G
L
N
K
M
N
K
T
L
I
A
L
A
V
S
A
A
A
V
A
T
G
A
Y
A
D
G
I
N
Q
S
G
D
K
A
G
S
T
V
Y
S
A
K
G
T
S
L
E
V
G
G
R
A
E
A
R
L
S
L
K
D
G
K
A
Q
D
N
S
R
V
R
L
N
F
L
G
K
A
E
I
N
D
S
L
Y
G
V
G
F
Y
E
G
E
F
T
T
N
D
Q
G
K
N
A
S
-
-
N
N
S
-
-
L
D
.
.
.
.
.
.
.
.
.
.
.
.
.
.
.
.
.
.
.
.
.
.
.
.
.
.
.
.
.
.
.
.
.
.
.
.
.
.
.
.
.
.
.
.
.
.
.
.
.
.
.
.
.
.
.
.
.
.
.
.
.
.
.
.
.
.
.
.
.
.
.
.
.
.
.
.
.
.
.
.
.
.
.
.
.
.
.
.
.
.
.
A
.
N
.
T
K
D
N
-
-
K
G
.
-
-
.
.
.
.
.
.
.
.
.
.
.
.
.
.
.
.
.
.
.
.
.
.
.
.
.
.
.
.
.
.
.
.
.
.
.
.
.
.
.
.
.
.
.
.
.
.
.
.
.
.
.
.
.
.
.
.
.
.
.
.
.
.
.
.
.
.
.
.
.
.
.
.
.
.
.
.
.
.
.
.
.
.
.
.
.
.
.
.
.
.
.
.
.
.
.
.
.
.
.
.
.
.
.
.
.
.
.
.
.
.
.
.
.
.
.
.
.
.
.
.
.
-
-
.
.
.
-
-
.
.
.
.
.
.
.
.
.
.
.
.
.
.
.
.
.
.
.
.
.
.
.
.
.
.
.
.
.
.
.
.
.
.
.
.
.
.
.
.
.
.
.
.
.
.
.
.
.
.
.
.
.
.
.
I
.
.
.
.
.
.
.
.
.
.
.
.
.
.
.
E
.
K
.
.
.
.
.
.
.
.
.
.
.
.
.
.
.
.
.
.
.
I
.
.
.
.
.
.
.
.
.
A
.
S
T
A
T
K
T
-
-
D
.
G
S
D
.
.
.
.
.
.
.
.
.
.
.
.
.
.
.
.
.
.
.
.
.
.
.
.
.
.
.
.
.
.
.
.
.
.
.
.
.
.
.
.
.
.
.
.
.
.
.
.
.
.
.
.
.
.
.
I
.
.
.
.
.
.
.
.
.
.
.
.
.
.
S
E
.
K
.
.
.
.
.
.
.
.
.
.
.
.
.
.
.
.
.
.
.
I
.
.
.
.
.
.
.
.
.
A
.
N
T
-
-
-
-
-
-
-
D
G
S
E
.
.
.
.
.
.
.
.
.
.
.
.
.
.
.
.
.
.
.
.
.
.
.
.
.
.
.
.
.
.
.
.
.
.
.
.
.
.
.
.
.
.
.
.
.
.
.
.
.
.
.
.
.
.
.
.
.
.
.
.
.
.
.
.
.
.
.
.
.
.
.
.
.
.
.
.
.
.
.
.
.
.
.
.
.
.
.
.
.
.
.
.
.
.
.
.
.
.
.
.
.
.
.
.
.
.
.
.
.
E
.
-
-
.
.
.
-
-
.
.
.
.
.
.
.
.
.
.
.
.
.
.
.
.
.
.
.
.
.
.
.
.
.
.
.
.
.
.
.
.
.
.
.
.
.
.
.
.
.
.
.
.
.
.
.
.
.
.
.
.
.
.
.
I
.
.
.
.
.
.
.
.
.
.
.
.
.
.
.
E
.
K
.
.
.
.
.
.
.
.
.
.
.
.
.
.
.
.
.
.
.
I
.
.
.
.
.
.
.
.
.
A
.
S
T
A
T
K
T
-
-
D
.
G
S
D
.
.
.
.
.
.
.
.
.
.
.
.
.
.
.
.
.
.
.
.
.
.
.
.
.
.
.
.
.
V
N
.
-
-
-
-
-
-
.
E
-
-
-
-
-
I
.
N
Q
D
.
A
.
.
T
M
.
.
.
.
.
.
.
.
.
.
.
.
.
.
.
E
.
K
.
.
.
.
.
.
.
.
.
.
.
.
.
.
.
.
.
.
.
.
.
.
.
.
.
.
.
.
.
.
.
S
V
S
Y
E
D
P
E
K
.
G
S
D
.
.
.
.
.
.
.
.
.
.
.
.
.
.
.
.
.
.
.
.
.
.
.
.
.
.
.
.
.
V
N
.
-
-
-
-
-
-
.
E
-
-
-
-
-
I
.
N
Q
D
.
A
.
.
T
M
.
.
.
.
.
.
.
.
.
.
Q
.
.
.
.
D
.
K
.
.
.
.
.
.
.
.
.
.
V
A
.
.
.
.
.
.
.
.
.
.
.
.
.
Q
.
E
.
.
.
D
.
V
.
K
T
-
-
.
.
-
-
N
.
.
.
.
.
.
.
.
.
.
.
.
.
.
.
.
.
.
.
.
.
.
.
.
.
.
.
.
.
V
N
.
-
-
-
-
-
-
.
E
-
-
-
-
-
I
.
N
K
D
.
A
.
.
T
M
.
.
.
.
.
.
.
.
.
.
Q
.
.
.
.
D
.
K
.
.
.
.
.
.
.
.
.
.
V
A
.
.
.
.
.
.
.
.
.
.
.
.
.
.
.
.
.
A
.
N
T
D
G
-
-
-
-
-
S
-
-
E
.
.
.
.
.
.
.
.
.
.
D
.
K
.
.
.
.
.
.
.
.
.
.
V
A
.
.
.
.
.
.
.
.
.
.
.
.
.
.
.
.
.
A
.
K
.
T
T
D
E
-
-
K
G
-
-
S
.
.
.
.
.
.
.
.
.
.
.
.
.
.
.
.
.
.
.
.
.
.
.
.
.
.
.
.
.
V
N
.
-
-
-
-
-
-
.
E
-
-
-
-
-
I
.
N
Q
D
.
A
.
.
N
M
.
.
.
.
.
.
.
.
.
.
.
.
.
.
.
D
.
K
.
.
.
.
.
.
.
.
.
.
V
A
.
.
.
.
.
.
.
.
.
.
.
.
.
.
.
.
.
.
.
.
.
E
A
K
-
-
-
D
G
-
-
N
.
N
.
.
.
.
.
.
.
.
.
.
.
.
.
.
.
.
.
.
.
.
.
.
.
.
.
.
.
.
.
.
V
N
.
-
-
-
-
-
-
.
E
-
-
-
-
-
I
.
N
Q
D
.
A
.
.
T
M
.
.
.
.
.
.
.
.
.
.
.
.
.
.
.
.
.
.
.
.
.
.
.
.
.
.
.
.
.
.
.
V
N
.
-
-
-
-
-
-
.
E
-
-
-
-
-
I
.
N
Q
D
.
A
.
.
N
M
.
.
.
.
.
.
.
.
.
.
.
.
.
.
.
D
.
K
.
.
.
.
.
.
.
.
.
.
V
A
.
.
.
.
.
.
.
.
.
.
.
.
.
.
.
.
.
.
.
R
-
D
G
Q
-
-
-
.
.
-
-
N
.
E
.
.
.
.
.
.
.
.
.
.
.
.
.
D
.
K
.
.
.
.
.
.
.
.
.
.
V
A
.
.
.
.
.
.
.
.
.
.
.
.
.
.
.
.
.
A
.
S
T
A
T
E
T
-
-
D
.
G
S
D
.
.
.
.
.
.
.
.
.
.
.
.
.
.
.
.
.
.
.
.
.
.
.
.
.
.
.
.
.
V
N
.
-
-
-
-
-
-
.
E
-
-
-
-
-
I
.
N
Q
D
.
A
.
.
T
M
.
.
.
.
.
.
.
.
.
.
.
.
.
.
.
D
.
K
.
.
.
.
.
.
.
.
.
.
V
A
.
.
.
.
.
.
.
.
.
.
.
.
.
.
.
.
.
.
.
.
.
E
A
K
D
-
-
G
.
-
-
-
.
N
.
.
.
.
.
.
.
.
.
.
.
.
.
.
.
.
.
.
.
.
.
.
.
.
.
.
.
V
N
.
-
-
-
-
-
-
.
E
-
-
-
-
-
I
.
N
Q
D
.
A
.
.
T
M
.
.
3
4
N
R
Y
T
Y
A
G
I
G
G
T
Y
G
E
V
T
Y
G
K
N
D
G
A
L
G
V
I
T
D
F
T
D
I
M
S
Y
H
G
N
T
A
A
E
-
-
K
I
A
V
A
D
R
V
D
N
M
L
A
Y
K
G
Q
F
G
D
L
G
V
K
A
S
Y
R
F
A
D
R
N
A
V
D
A
M
G
N
V
V
T
E
T
N
A
A
K
Y
S
D
N
G
E
D
G
Y
S
L
S
A
I
Y
T
F
G
D
T
G
F
N
V
.
.
.
.
.
.
.
.
.
.
.
.
.
.
.
.
.
.
A
.
.
.
.
.
.
.
.
.
.
.
.
.
.
.
.
.
.
.
.
.
.
.
.
.
.
.
.
.
.
.
.
.
.
.
.
.
.
.
.
.
.
.
.
.
.
.
.
.
.
.
.
.
.
.
.
.
.
.
.
.
.
.
.
.
.
.
.
.
S
.
.
D
-
-
.
.
.
.
.
.
.
.
.
.
.
.
.
.
.
.
.
.
.
.
.
.
.
.
.
.
F
.
.
.
.
.
T
E
-
-
-
-
-
.
T
.
-
-
.
D
-
-
.
.
E
.
.
.
K
.
.
.
.
.
.
.
.
.
A
.
.
.
.
.
.
.
.
.
.
.
.
.
.
.
.
.
.
.
.
.
.
.
.
.
.
.
.
.
.
.
.
.
.
.
.
.
.
.
.
.
.
.
.
.
.
.
.
.
.
.
.
.
.
.
.
.
.
.
.
.
.
.
.
.
.
.
.
.
.
.
-
-
.
.
.
.
.
.
.
.
.
.
.
.
.
.
.
.
.
.
.
.
.
.
.
.
.
.
.
.
.
.
.
.
.
.
.
.
.
.
.
.
.
.
.
S
.
.
D
-
-
.
.
.
.
.
.
.
.
.
.
.
.
.
.
.
.
.
.
.
.
.
.
.
.
.
.
F
.
.
.
.
.
T
E
-
-
-
-
-
.
T
.
-
-
.
D
-
-
.
.
E
.
.
.
K
.
.
.
.
.
.
.
.
.
A
.
.
.
.
.
.
.
.
.
.
.
.
.
.
.
.
.
.
.
.
.
.
.
.
.
.
.
.
.
.
.
.
.
.
.
.
.
.
.
.
.
.
.
.
.
.
.
S
.
.
D
-
-
.
.
.
.
.
.
.
T
.
.
.
.
.
.
.
.
.
.
.
.
.
.
.
.
.
.
.
.
.
.
.
.
-
-
-
-
-
-
-
-
-
-
-
D
.
S
T
G
E
F
T
.
.
K
.
.
.
.
.
.
.
.
.
.
A
.
.
.
.
.
.
.
I
.
.
.
.
.
.
.
.
.
.
.
.
.
.
.
.
.
.
.
.
.
.
.
.
.
.
.
.
.
.
.
.
.
.
.
.
.
.
.
S
.
.
D
-
-
.
.
.
.
.
.
.
.
.
.
.
.
.
.
.
.
.
.
.
.
.
.
.
.
.
.
.
.
.
.
.
.
V
E
-
-
-
-
-
G
T
G
-
-
.
.
S
G
Q
.
V
.
.
.
K
.
.
.
.
.
.
.
.
.
A
.
.
.
.
.
.
.
.
.
.
.
.
.
.
.
.
.
.
.
.
.
.
.
.
.
.
.
.
.
.
.
.
.
.
.
.
.
.
.
.
.
.
.
.
.
.
.
S
.
.
D
-
-
.
.
.
.
.
.
.
.
.
.
.
.
.
.
.
.
.
.
.
.
.
.
.
.
.
.
F
.
.
.
.
.
T
E
-
-
-
-
-
.
T
.
-
-
.
D
-
-
.
.
E
.
.
.
K
.
.
.
.
.
.
.
.
.
A
.
.
.
.
.
.
.
.
.
.
.
.
.
.
.
.
.
.
.
.
.
.
.
.
.
.
.
.
.
.
.
.
.
.
.
.
.
.
.
.
.
.
A
.
.
.
.
S
.
.
D
-
-
.
.
.
.
.
.
.
T
.
.
.
.
.
.
.
.
.
.
.
.
.
.
.
.
.
.
F
.
.
.
.
.
K
E
N
K
.
-
-
-
-
-
-
-
.
D
-
-
.
F
E
.
.
.
K
.
.
.
.
.
.
.
.
.
A
.
.
.
.
.
.
S
.
.
.
.
.
.
.
.
.
.
.
.
.
.
.
.
.
.
.
.
.
.
.
.
.
.
.
.
.
.
.
.
.
.
.
A
.
.
.
.
S
.
.
-
-
Y
.
.
.
.
.
.
.
.
.
.
.
.
.
.
.
.
.
.
.
.
.
.
.
.
.
.
.
.
.
.
.
.
K
D
G
-
T
G
S
.
-
-
-
A
-
-
-
G
.
F
V
.
.
D
K
.
.
.
.
.
.
.
.
.
A
.
.
.
.
.
.
.
I
.
.
.
.
.
.
.
.
.
.
.
.
.
.
.
.
.
.
.
.
.
.
.
.
.
.
.
.
.
.
.
.
.
.
A
.
.
.
A
.
.
V
G
E
S
.
L
P
.
.
.
.
T
.
.
.
.
.
.
.
.
.
.
.
.
.
.
.
.
.
.
.
.
.
.
.
.
-
-
-
-
K
N
V
A
-
-
-
G
-
-
-
-
.
.
E
.
.
.
L
.
.
.
.
.
.
.
V
.
A
I
.
E
.
.
I
T
L
.
.
.
.
.
.
.
.
.
.
.
.
.
.
.
.
.
.
.
.
.
.
.
.
.
.
.
.
.
.
.
.
.
.
A
.
.
.
.
S
.
.
-
-
Y
.
.
.
.
.
.
.
.
.
.
.
.
.
.
.
.
.
.
.
.
.
.
.
.
.
.
.
.
.
.
.
.
S
.
D
-
K
D
V
.
-
-
-
G
D
W
N
N
A
.
T
.
.
N
A
.
.
.
.
.
.
.
V
.
A
I
.
E
.
.
I
T
L
.
.
.
.
.
.
.
.
.
.
.
.
.
.
.
.
.
.
.
.
.
.
.
.
.
.
.
.
.
.
.
.
.
.
A
.
.
.
.
S
.
.
-
-
Y
.
.
.
.
.
.
.
.
.
.
.
.
.
.
.
.
.
.
.
.
.
.
.
.
.
.
.
.
.
.
.
.
S
.
D
-
K
N
A
.
-
-
-
G
D
W
N
N
A
.
T
.
.
N
A
.
.
.
.
.
.
.
V
.
A
.
.
.
.
.
.
.
I
.
.
.
.
.
.
.
.
.
.
.
.
.
.
.
.
.
.
.
.
.
.
.
.
.
.
.
.
.
.
.
.
.
.
A
.
.
.
A
.
.
V
G
E
S
.
L
H
.
.
.
.
A
.
.
.
.
.
.
.
.
.
.
.
.
.
.
.
.
.
.
.
.
.
.
.
.
-
-
D
-
V
N
A
G
-
-
-
-
-
-
-
-
.
F
V
.
.
N
K
.
.
.
.
.
.
.
V
.
A
.
.
.
.
.
.
.
I
.
.
.
.
.
.
.
.
.
.
.
.
.
.
.
.
.
.
.
.
.
.
.
.
.
.
.
.
.
.
.
.
.
.
A
.
.
.
E
N
G
T
-
-
Y
.
L
.
.
.
.
.
.
.
.
.
.
.
.
.
.
.
.
.
.
.
.
.
.
.
.
.
.
.
.
.
.
D
E
D
G
K
F
V
E
-
-
-
G
V
S
N
D
.
F
I
.
K
D
N
.
.
.
.
.
.
.
.
.
.
.
.
.
.
.
.
.
.
.
.
.
.
.
.
.
.
.
.
.
.
.
.
.
.
.
.
.
.
.
.
.
.
.
.
.
.
.
.
.
.
.
.
A
.
.
.
A
N
G
T
-
-
Y
.
L
.
.
.
.
.
.
.
.
.
.
.
.
N
.
.
.
.
.
.
.
.
.
.
.
.
.
.
.
.
.
Q
E
S
-
-
-
V
.
-
-
-
S
I
T
K
N
-
-
-
-
-
E
Q
.
.
.
.
.
.
.
.
.
A
.
.
.
.
.
.
.
.
5
6
G
A
G
Y
A
D
Q
D
D
Q
-
-
-
-
N
E
Y
M
L
A
A
S
Y
R
M
E
N
L
Y
F
A
G
L
F
T
D
G
E
L
-
-
-
A
K
-
D
V
-
-
-
-
-
-
-
-
-
-
-
-
-
D
Y
T
G
Y
E
L
A
A
G
Y
K
L
G
Q
A
A
F
T
A
T
Y
N
N
A
E
T
-
-
-
-
A
K
E
T
S
A
D
N
F
A
I
D
A
T
Y
Y
F
K
P
N
F
R
S
Y
I
S
Y
.
.
.
.
.
.
.
.
.
.
.
.
.
.
.
.
.
.
.
.
.
.
.
.
.
E
.
-
-
-
-
.
.
.
.
.
.
.
.
.
.
.
.
.
.
.
.
.
.
.
.
.
.
.
.
K
-
-
-
.
.
-
.
.
-
-
-
-
-
-
-
-
-
-
-
-
-
.
.
.
.
.
.
.
.
.
.
.
.
.
.
.
.
.
.
.
.
.
.
.
.
.
.
.
-
-
-
-
.
.
.
.
.
.
.
.
.
.
.
.
.
.
.
.
.
.
.
.
.
.
.
.
.
.
.
.
.
.
.
.
.
.
.
E
.
-
-
-
-
.
.
.
.
.
.
.
.
.
.
.
.
.
.
.
.
.
.
.
.
.
.
.
.
.
.
.
.
E
.
-
-
-
-
.
.
.
.
.
.
.
.
.
.
.
.
.
.
.
.
.
.
.
.
.
.
.
.
.
-
-
-
.
.
-
.
.
-
-
-
-
-
-
-
-
-
-
-
-
-
.
.
.
.
.
.
.
.
.
.
.
.
.
.
.
.
.
.
.
.
.
.
.
.
.
.
.
-
-
-
-
.
.
.
.
.
.
.
.
.
.
.
.
.
.
K
-
-
-
.
.
-
.
.
-
-
-
-
-
-
-
-
-
-
-
-
-
.
.
.
.
.
.
.
.
.
.
.
.
.
.
.
.
.
.
.
.
.
.
.
.
.
.
.
-
-
-
-
.
.
.
.
.
.
.
.
.
.
.
.
.
.
.
.
.
.
.
.
.
.
.
.
.
.
.
.
.
.
.
.
.
.
N
.
N
-
-
-
-
.
.
.
.
.
.
.
.
.
.
.
.
.
V
.
.
G
A
.
.
.
.
.
.
K
N
F
N
S
.
S
N
G
N
N
S
V
V
K
G
K
F
T
G
I
Q
.
.
.
.
.
.
.
.
.
.
.
.
.
.
.
.
.
.
.
T
.
.
.
.
.
.
.
-
-
-
-
.
N
D
.
.
.
N
.
V
.
.
.
.
.
.
.
.
.
.
.
.
.
T
.
.
.
.
.
.
.
.
.
.
.
.
E
.
-
-
-
-
.
.
.
.
.
.
.
.
.
.
.
.
.
.
.
.
.
.
.
.
.
.
.
.
K
-
-
-
.
A
T
N
G
-
-
-
-
-
-
-
-
-
-
-
-
-
.
.
.
.
.
.
.
.
.
.
.
.
.
.
.
.
.
.
.
T
.
.
.
.
.
.
.
-
-
-
-
N
D
.
.
.
.
N
.
.
.
.
.
.
.
.
.
.
.
.
.
.
.
T
.
.
.
.
.
.
.
.
.
.
.
.
E
.
-
-
-
-
.
.
.
.
.
.
.
.
.
.
.
.
.
.
.
.
.
.
.
.
.
.
.
.
K
-
-
-
.
A
T
N
G
-
-
-
-
-
-
-
-
-
-
-
-
-
.
.
.
.
.
.
.
.
.
.
.
.
.
.
.
.
.
.
.
T
.
.
.
.
.
.
.
-
-
-
-
N
D
.
.
.
.
N
.
.
.
.
.
.
.
.
.
.
.
.
.
.
.
T
.
.
.
.
.
.
.
.
.
.
.
.
T
.
-
-
-
-
D
Q
.
.
.
.
.
.
.
.
.
.
.
.
.
.
.
.
.
.
.
.
.
.
K
-
-
-
.
A
T
N
G
-
-
-
-
-
-
-
-
-
-
-
-
-
.
.
.
.
.
.
.
.
.
.
.
.
.
.
.
.
.
.
.
T
.
.
.
.
.
.
.
-
-
-
-
N
D
.
.
.
T
N
K
.
.
.
.
.
.
.
.
.
.
.
.
.
.
.
.
.
.
.
.
.
.
.
.
.
.
.
T
.
-
-
-
-
D
Q
.
.
.
.
.
.
.
A
I
A
D
F
.
.
.
.
S
.
V
.
.
Q
E
K
P
A
N
A
A
K
T
-
-
-
-
-
-
-
-
-
-
-
-
-
.
K
.
.
.
.
.
.
.
K
.
T
M
.
.
.
V
.
S
T
.
.
.
Y
L
.
S
K
S
S
G
T
.
T
D
E
.
.
.
.
.
.
.
.
.
.
.
.
.
.
.
.
.
.
.
.
.
.
.
.
.
.
.
.
.
E
T
.
-
-
-
-
S
Q
.
.
.
.
.
.
.
V
I
S
D
F
.
.
.
.
S
.
V
.
.
Q
D
K
P
A
N
Q
T
K
A
-
-
-
-
-
-
-
-
-
-
-
-
-
.
L
.
.
.
.
.
.
.
K
.
T
M
.
.
.
V
.
S
T
.
.
.
Y
L
.
S
K
T
S
G
T
.
A
D
E
.
.
.
.
.
.
.
.
.
.
.
.
.
.
.
.
.
.
.
.
.
.
.
.
.
.
.
.
.
.
N
.
N
T
S
A
D
Q
.
.
.
.
.
.
.
T
I
S
D
F
.
.
.
.
T
.
V
.
.
Q
V
K
E
F
N
D
-
K
A
-
-
-
-
-
-
-
-
-
-
-
-
-
.
L
.
.
.
.
.
.
.
K
.
T
M
.
.
.
V
.
S
T
.
.
.
Y
L
.
S
K
S
S
G
T
.
T
D
E
.
.
.
.
.
.
.
.
.
.
.
.
.
.
.
.
.
.
.
.
.
.
.
.
.
.
.
.
.
.
T
E
-
-
-
-
D
Q
.
.
.
.
.
.
.
A
I
A
D
F
.
.
.
.
A
.
V
.
.
Q
D
K
P
A
N
A
A
K
T
-
-
-
-
-
-
-
-
-
-
-
-
-
.
K
.
.
.
.
.
.
.
K
.
T
M
.
.
.
V
.
S
T
.
.
.
Y
L
.
S
K
S
S
G
T
.
T
D
E
.
.
.
.
.
.
.
.
.
.
.
.
.
.
.
.
.
.
.
.
.
.
.
.
.
.
.
.
.
.
T
.
-
-
-
-
D
Q
.
.
.
.
.
.
.
A
I
A
D
F
.
.
.
.
S
.
V
.
.
Q
D
K
P
A
N
A
A
K
T
-
-
-
-
-
-
-
-
-
-
-
-
-
.
K
.
.
.
.
.
.
.
K
.
T
M
.
.
.
V
.
S
T
.
.
.
Y
L
.
S
K
S
S
G
T
.
T
D
E
.
.
.
.
.
.
.
.
.
.
.
.
.
.
.
.
.
.
.
.
.
.
.
.
.
.
.
.
.
.
T
-
-
-
-
A
D
Q
.
I
.
.
.
.
.
A
F
S
D
F
.
.
.
.
A
.
V
.
.
Q
V
K
P
A
N
A
A
K
R
-
-
-
-
-
-
-
-
-
-
-
-
-
.
K
.
.
.
.
.
.
.
K
.
T
M
D
.
T
V
.
.
T
.
.
.
Y
Q
.
E
K
T
S
G
V
.
S
D
N
.
.
F
I
.
.
.
.
.
.
.
.
.
.
.
.
.
.
.
V
.
.
.
.
.
.
.
.
.
.
T
-
-
-
-
A
D
Q
.
I
.
.
.
.
.
A
F
S
D
F
.
.
.
.
A
.
V
.
.
Q
V
K
P
A
N
A
A
K
T
-
-
-
-
-
-
-
-
-
-
-
-
-
.
K
.
.
.
.
.
.
.
K
.
T
M
D
.
T
V
.
.
T
.
.
.
Y
Q
.
E
K
T
S
G
V
.
S
D
N
.
.
F
I
.
.
.
.
.
.
.
.
.
.
.
.
.
.
.
V
.
.
7
Q
F
N
L
L
D
S
D
-
-
-
-
K
V
G
-
-
-
-
-
-
K
V
A
S
E
D
E
L
A
I
G
L
R
Y
D
F
.
.
.
.
.
.
.
.
-
-
-
-
.
.
.
-
-
-
-
-
-
.
.
.
.
.
.
.
.
.
.
.
.
.
.
.
.
N
.
.
.
.
.
.
.
-
-
-
-
.
.
.
-
-
-
-
-
-
.
.
.
.
.
.
.
.
.
.
.
.
.
.
.
.
N
.
.
.
.
.
N
.
.
.
.
.
.
G
D
-
-
-
.
L
.
-
N
S
T
V
S
.
I
D
A
.
.
.
.
.
.
.
.
.
.
.
.
N
.
.
M
I
.
A
G
D
V
L
G
.
.
.
G
N
G
V
A
T
.
I
D
A
.
.
.
.
.
.
.
.
.
.
.
.
N
.
.
M
I
.
A
G
D
V
L
G
.
.
.
G
N
G
V
A
T
.
I
D
A
.
.
.
.
.
.
.
.
.
.
.
.
N
.
.
M
I
.
A
G
D
V
L
G
.
.
.
G
N
G
V
A
T
.
I
D
A
.
.
.
.
.
.
.
.
.
.
.
.
N
.
.
.
.
.
E
.
-
-
-
-
-
-
-
A
N
K
G
I
T
.
A
Q
A
.
.
.
.
.
.
.
.
.
.
.
.
.
-
-
-
-
.
.
.
-
-
-
-
-
-
.
.
.
.
.
.
.
.
.
.
.
C
l
u
s
t
e
r
1
C
l
u
s
t
e
r
2
.
.
.
.
.
.
N
.
.
.
.
.
E
.
-
-
-
-
-
-
-
A
N
K
G
I
T
.
A
Q
A
.
.
.
.
.
.
.
.
.
.
.
.
N
.
.
.
.
.
E
.
-
-
-
-
-
-
-
A
N
K
G
I
T
.
A
Q
A
.
.
.
.
.
.
.
.
.
.
.
.
N
.
.
.
.
.
E
.
-
-
-
-
-
-
-
A
N
K
G
I
T
.
A
Q
A
.
.
.
.
.
.
.
.
.
.
.
.
N
.
.
.
.
.
E
.
-
-
-
-
-
-
-
A
N
K
G
I
T
.
A
Q
V
.
.
.
.
.
.
.
.
.
.
.
.
N
.
.
.
.
.
A
.
-
-
-
-
-
-
-
K
-
-
-
V
G
.
T
K
A
.
.
.
.
.
.
.
.
.
.
.
.
N
.
.
.
.
.
A
.
-
-
-
-
-
-
-
K
-
-
-
V
G
.
T
K
A
.
.
.
.
.
.
.
.
.
.
.
.
