# Supplementary figures and images for: Allelic diversity uncovers protein domains contributing to the emergence of antimicrobial resistance
Source: PLoS Genet. 2023 Mar 27;19(3):e1010490. doi: 10.1371/journal.pgen.1010490 (PMC10079234; doi:10.1371/journal.pgen.1010490)

## Slide 1
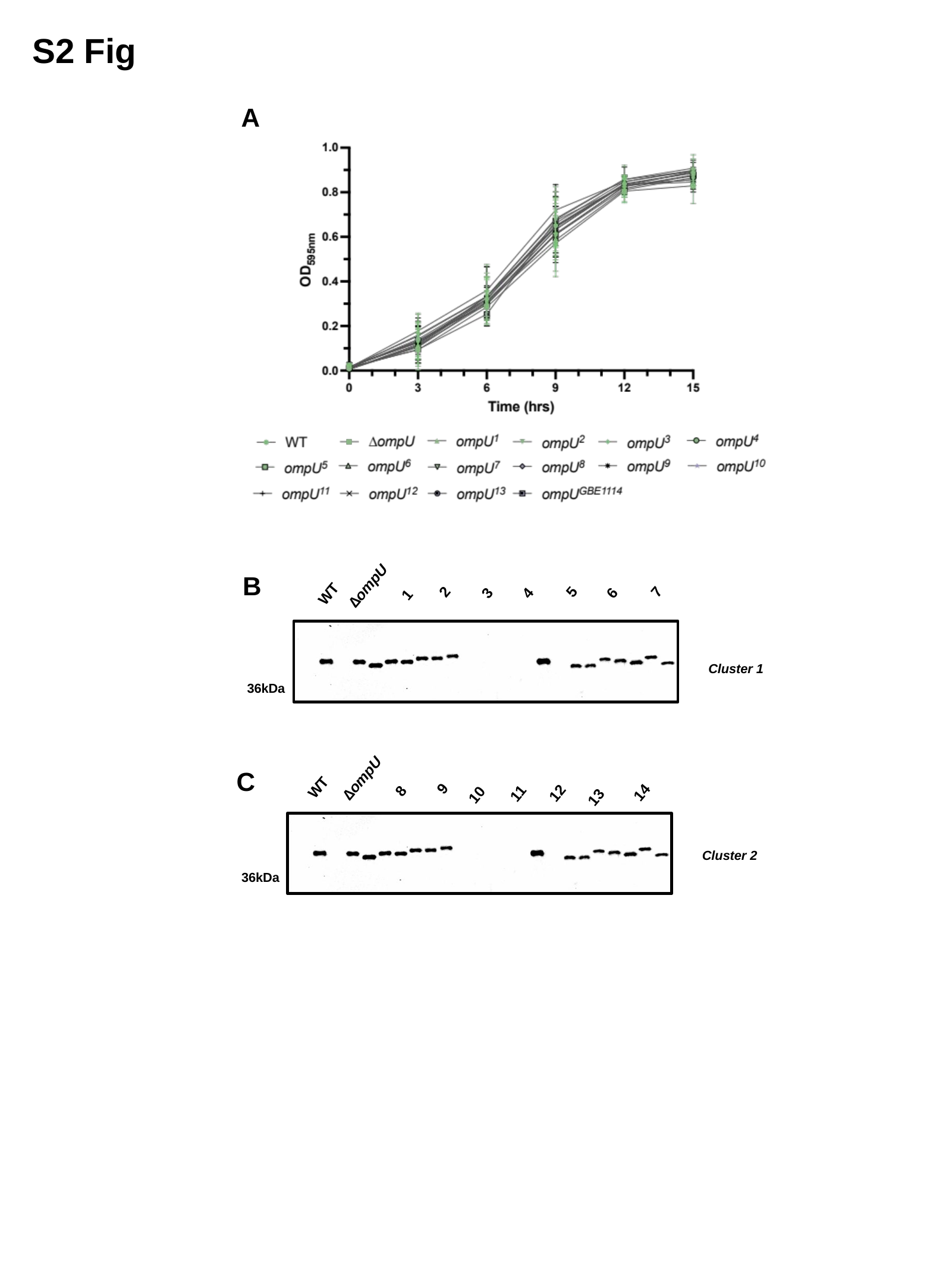

S2 Fig
A
∆ompU
B
WT
5
7
2
3
4
6
1
Cluster 1
36kDa
∆ompU
C
WT
9
12
14
11
13
8
10
Cluster 2
36kDa

Supplement: S2 Fig — (A) Growth analysis of isogenic mutant strains encoding environmental ompU alleles, N≥3. (B-C) Immunoblots of OmpU from representative clades. 200ng of whole cell lysates from strains encoding alleles from (B) Cluster 1 and (C) Cluster 2 were probed using α-OmpU antibodies. (PPTX) [file pgen.1010490.s004.pptx]

## Slide 1
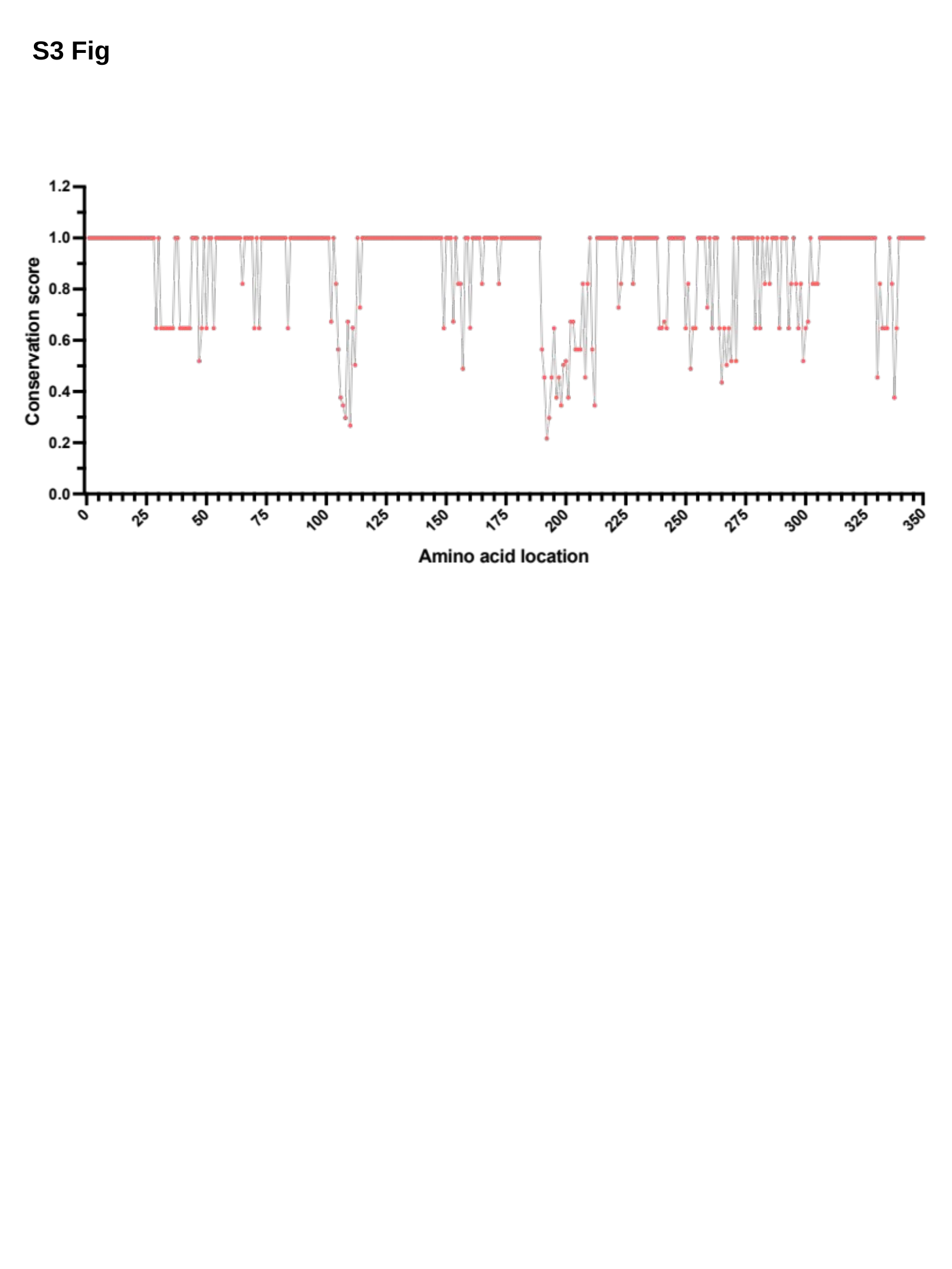

S3 Fig

Supplement: S3 Fig — Scorecons result for alignments of bile resistant and sensitive allele groups. Conservation scores (y-axis) range between 0 (not conserved) and 1 (conserved) and are indicated by the pink dots. Amino acid location (x-axis) is relative to the WT allele (N16961). (PPTX) [file pgen.1010490.s005.pptx]

## Slide 1
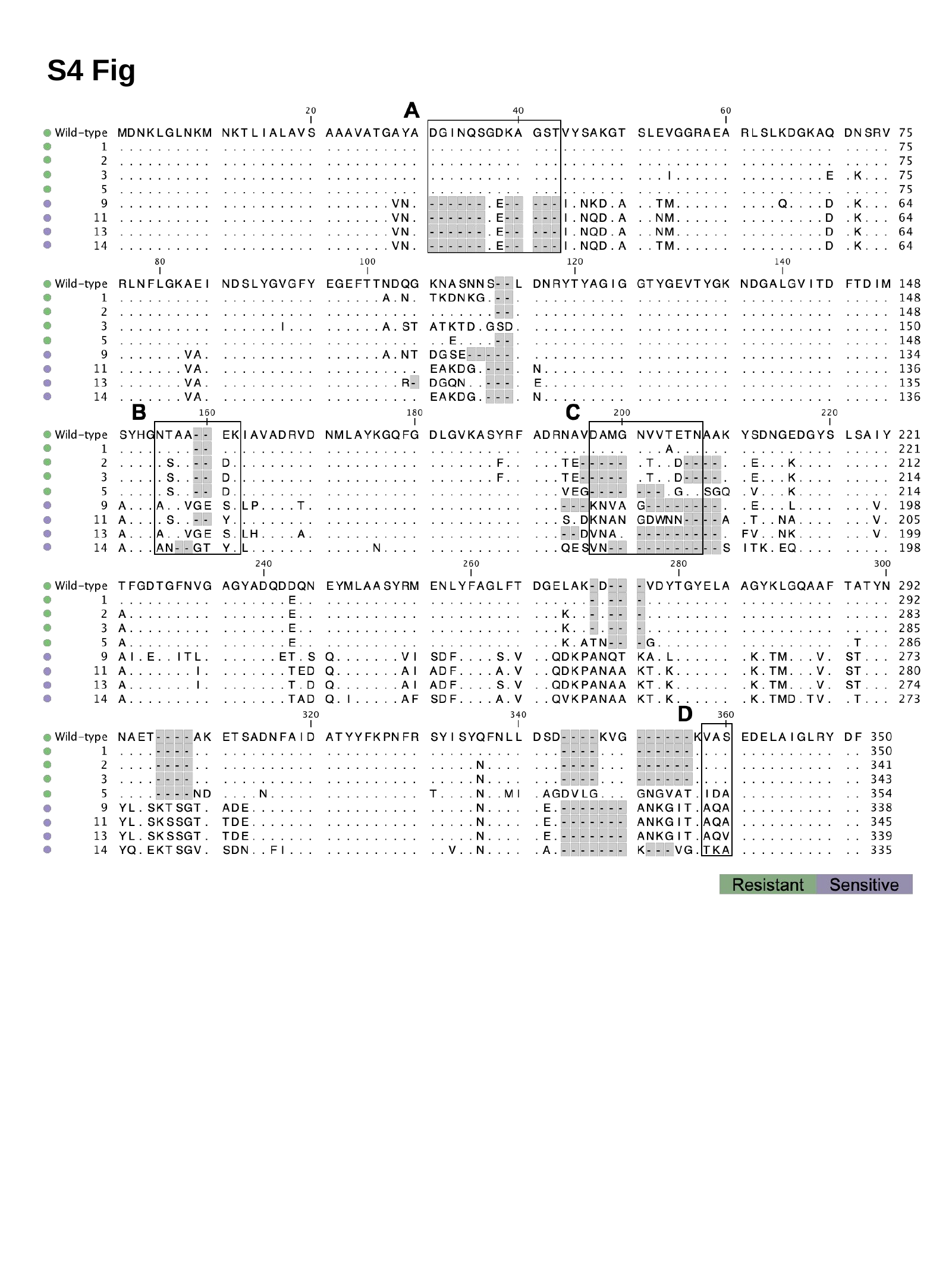

S4 Fig

Supplement: S4 Fig — Gray boxes represent missing residues and black dots represent conserved residues. Highlighted in black boxes are confirmed regions of interest lettered A-D. Green and purple dots represent alleles from Cluster 1 and Cluster 2, respectively. (PPTX) [file pgen.1010490.s006.pptx]

## Slide 1
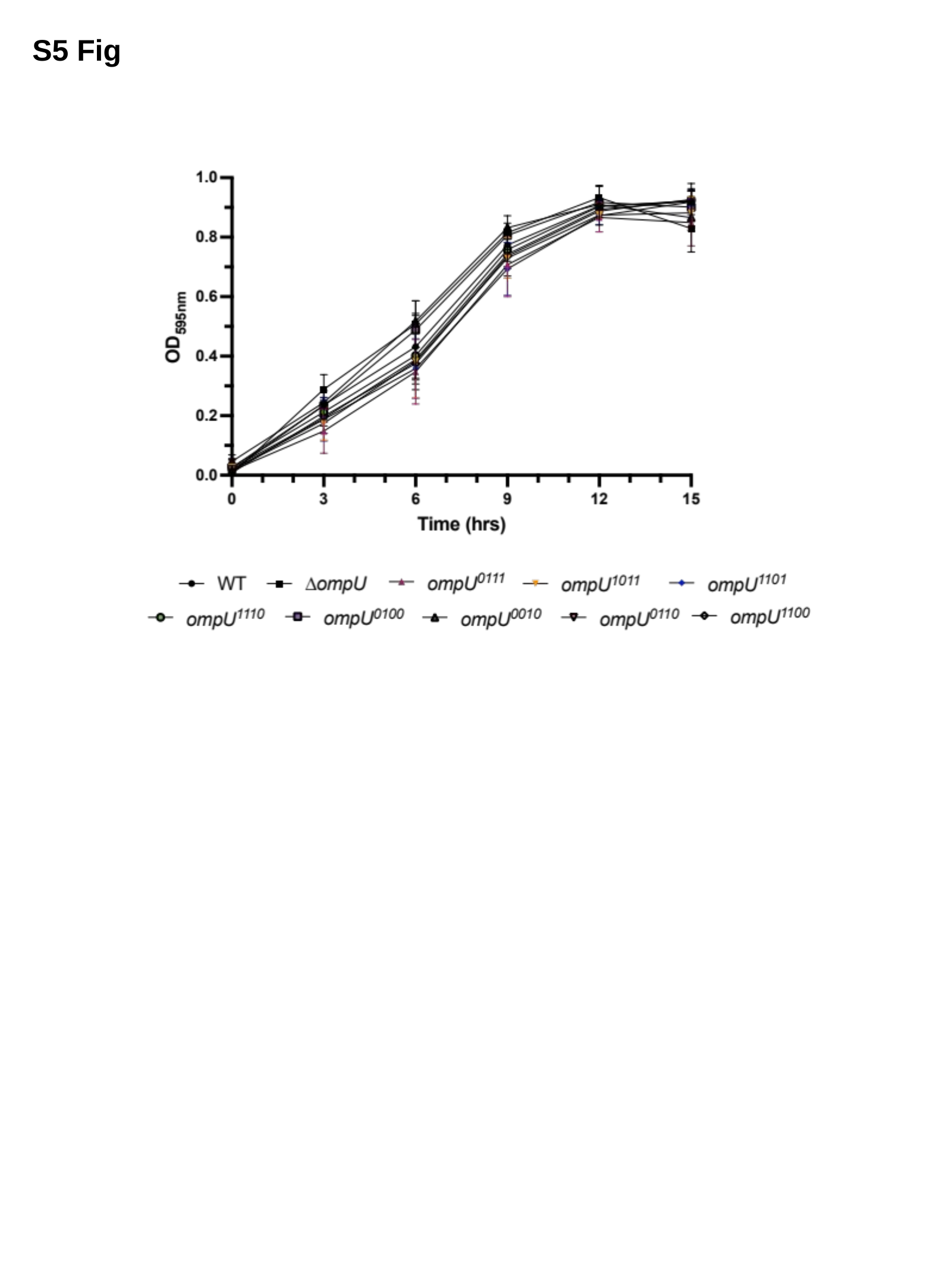

S5 Fig

Supplement: S5 Fig — Strains were cultured overnight in LB at 37°C with aeration using the Tecan Sunrise microplate reader (Tecan) and optical density (OD) was measured hourly at 595 nm using the Magellan plate reader software (Tecan), N≥3. (PPTX) [file pgen.1010490.s007.pptx]

## Slide 1
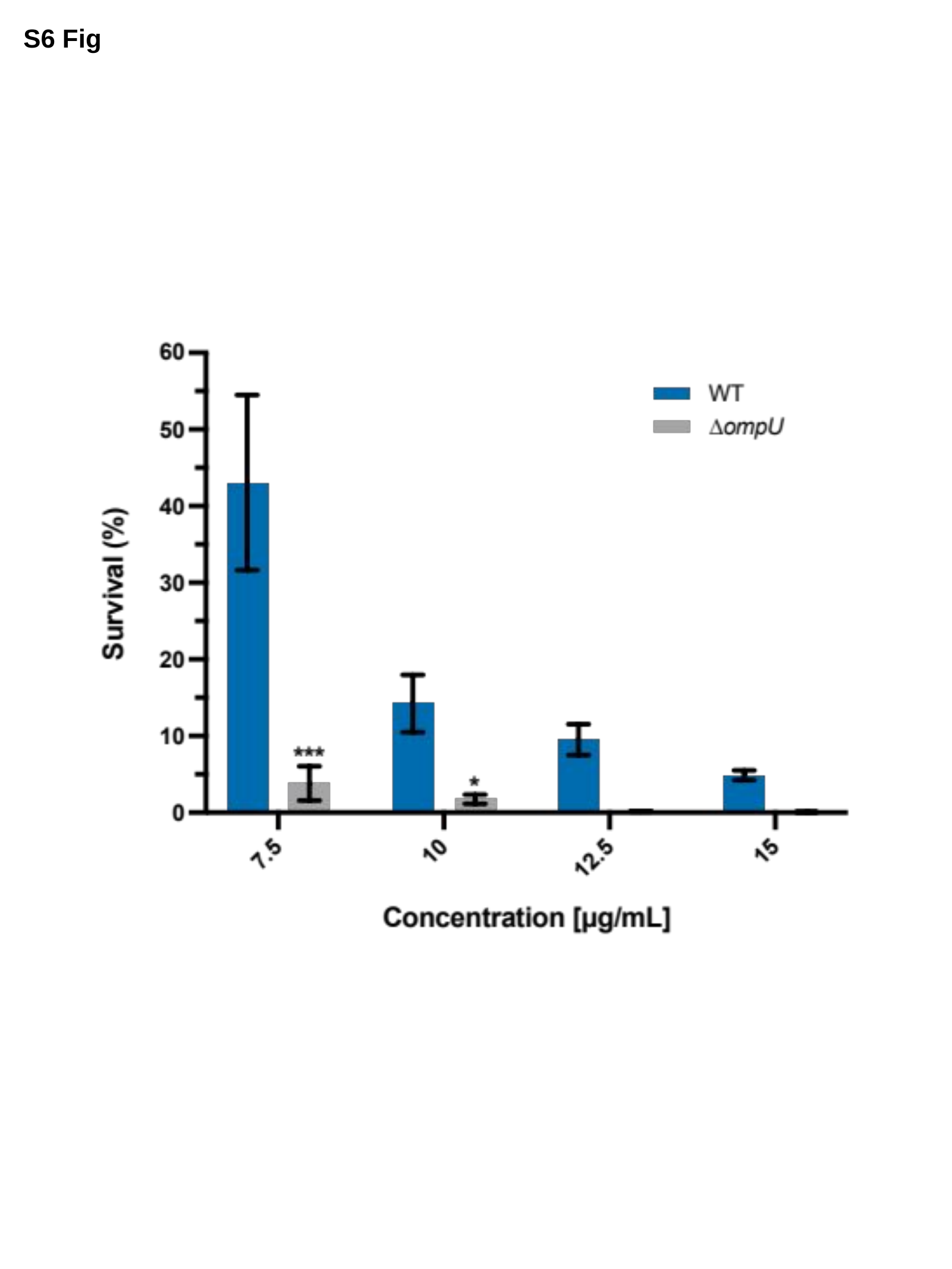

S6 Fig

Supplement: S6 Fig — Survival of V. cholerae N16961 WT and ΔompU in the presence of varying concentrations of rifamycin SV. Statistical comparisons were performed using two-way ANOVA analyses followed by a Bonferroni’s multiple comparison test. All constructs were compared to the WT unless otherwise stated, N≥3. *p<0.05, **p<0.01, ***p<0.001. (PPTX) [file pgen.1010490.s008.pptx]

## Slide 1
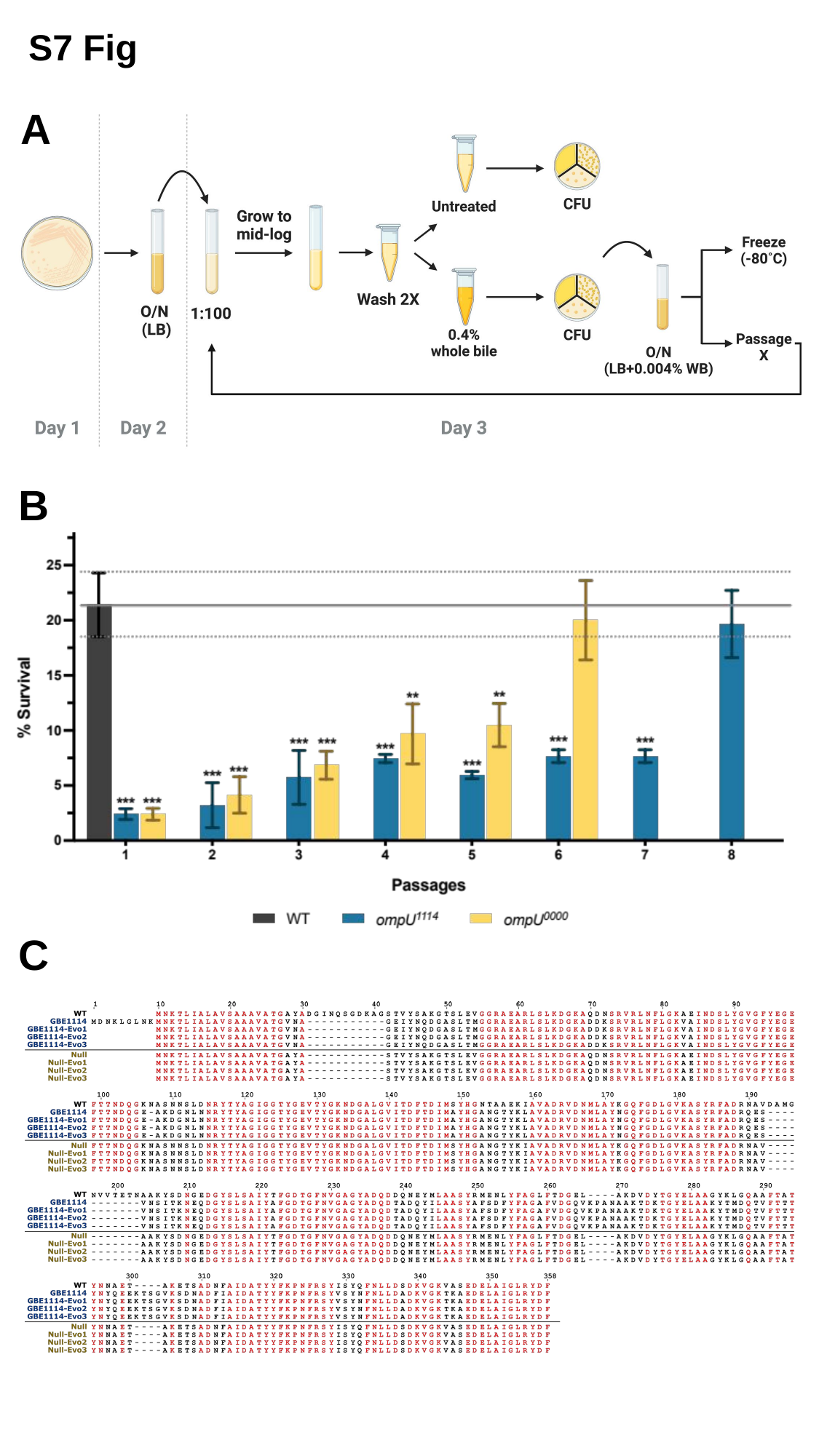

S7 Fig
A
B
C

Supplement: S7 Fig — (A) Schematic of the methodology for the in vitro evolution experiments of V. cholerae OmpU isogenic mutant strains in the presence of 0.4% whole bile (Adapted from Levin-Reisman et al [89]). (B) Survival of V. cholerae isogenic mutant strains encoding ompU alleles ompUGBE1114 and ompU0000 over successive passages through 0.4% whole bile. n = 3. Statistical comparisons were performed using one-way ANOVA analyses followed by a Dunnett’s multiple comparison test. All constructs were compared to the WT unless otherwise stated. *p<0.05, **p<0.01, ***p<0.001. (C) Alignment of OmpU variants. Wild-type N16961 (WT), OmpU0000 (Null), OmpU0000 after bile passages (Null-Evo1, Null-Evo2, Null-Evo3), OmpUGBE1114 (GBE1114), OmpUGBE1114 after bile passages (GBE1114-Evo1, GBE1114-Evo2, GBE1114-Evo3). (PPTX) [file pgen.1010490.s009.pptx]
